# Cross-Chip Probe Matching Tool: A Web-Based Tool for Linking Microarray Probes within and across Plant Species

**DOI:** 10.1155/2008/451327

**Published:** 2008-10-21

**Authors:** Ruchi Ghanekar, Vinodh Srinivasasainagendra, Grier P. Page

**Affiliations:** ^1^Department of Electrical and Computer Engineering, UAB School of Engineering, University of Alabama at Birmingham, 1530 Third Avenue South, Birmingham, AL 35294-4461, USA; ^2^Department of Biostatistics, University of Alabama at Birmingham, 1665 University Blvd, Birmingham, Al 35294-0022, USA; ^3^Statistics and Epidemiology Unit, RTI International, Oxford Building, Suite 119, 2951 Flowers Road South, Atlanta, GA 30341-5533, USA

## Abstract

The CCPMT is a free, web-based tool that allows plant investigators to rapidly determine if a given gene is present across various microarray platforms, which, of a list of genes, is present on array(s), and which gene a probe or probe set queries and vice versa, and to compare and contrast the gene contents of arrays. The CCPMT also maps a probe or probe sets to a gene or genes within and across species, and permits the mapping of the entire content from one array to another. By using the CCPMT, investigators will have a better understanding of the contents of arrays, a better ability to link data between experiments, ability to conduct meta-analysis and combine datasets, and an increased ability to conduct data mining projects.

## 1. INTRODUCTION

Microarrays are an incredibly powerful technology that enables the rapid and relatively accurate measurement of thousands of genes in a single sample. Many different microarray platforms have been developed and each has somewhat different content and format. One key difference is the type of probe used to query a gene expression; some platforms use a single probe, and others use many probes. The probes may be short (25 base pairs) oligonucleotides (Affymetrix and NimbleGen arrays), long (50–70 bp) oligonucleotides (Operon, Agilent, CATMA), or cDNA clones (AFGC arrays). Each of the formats has its advantages and disadvantages as well as its proponents and opponents. One thing on which everybody agrees is that arrays will be a part of the experimental techniques of plant biologists for years to come.

Since there are many microarray platforms even within a single species, different investigators may use different platforms to try to address similar or complementary experimental questions or data may be collected across types using different platforms. Also, the large number of datasets that sets in the public domain allow can be used for data mining or meta-analysis if the elements can be connected. However, it is difficult to compare and combine the results due to the difficultly in matching probes across arrays with the genes, or even to determine if a given gene is on a given platform. To make matters worse, while the probe sequences on an array are constant, the genome annotation and gene models are not, and homologous genes may have different names across species. As a result, matching probes across arrays is continually evolving and needs continuing updating.

Investigators have long realized the problem of linking probes across platforms; as a result, several tools have been developed. These include Keck ARray Manager and Annotator (KARMA) [1], RESOURCERER [2], and GeneSeer [3]. Our tool has several advantages over the other tools for several reasons. None of the other tools allows investigators to query for genes within a microarray platform nor do the other tools allow queries by *Arabidopsis* Genome Initiative (AGI) annotation IDs or by TIGR tentative consensus (TC) gene IDs. Furthermore, our tool sends the results to the investigators by email as well as a web-based report making results' tracking and storage easier. More importantly for plant researchers, only RESOURCERER has any provision for the linking of plant array data, but it has fewer array types.

We developed the CCPMT to enable investigators to rapidly determine (1) if a given gene is present across many types of array platforms within and across species, (2) which, of a list of genes, is present on array(s), and (3) which gene a probe or probe set queries. The CCPMT also maps a probe set or probe sets to a gene or genes within and across species, and permits the mapping of the contents from one array to another, both within and across species.

The CCPMT is the first tool exclusively designed for linking probes from plant microarrays within and across microarray platforms and species. A web-based tool, CCPMT, helps investigators query for annotations at probe level with probe set IDs or even at gene level with gene identifiers such as AGI, EGO [4], and TC IDs. In CCPMT, an investigator can enter either individual or multiple probe set or gene identifiers (separated by commas) in the textbox to query the CCPMT database. Checkboxes for microarray vendors provide the option of selecting multiple arrays while querying the CCPMT database. CCPMT also offers the flexibility to carry out a one-to-one comparison of microarrays. Results are displayed immediately in the web browser and are also sent through email in a ^*∗*^csv file format.

CCPMT has a flexible database design, and in the immediate future additional plant arrays will be added to the database; we will revise the underlying annotation and mapping for the probes based upon new genomic information.

By using the CCPMT, investigators will have a better understanding of the contents of arrays, a better ability to link data between experiments, plus the ability to more easily conduct data mining projects.

## 2. METHODS

### 2.1. Arrays selected for initial analysis

Initially we focused upon microarrays with diverse probe types (short and long oligos as well as cDNA) and for both *Poplar* and *Arabidopsis*. *Poplar* and *Arabidopsis* were chosen due to both having completely sequenced genomes and being relatively closely related species. The *Arabidopsis* arrays as tools are the Affymetrix *Arabidopsis* genome (8 K) commonly referred to as AG, Affymetrix *Arabidopsis* genome ATH1-121501 (25 K) commonly referred to as ATH1, Agilent *Arabidopsis* 2 Oligo Microarray (V2) G4136B, *Arabidopsis* Functional Genomics Consortium (AFGC) array, Complete *Arabidopsis* Transcriptome MicroArray (CATMA) array, Operon *Arabidopsis* Genome Oligo Set Version 3.0, and Affymetrix *Poplar* Genome Array. The array that we are calling AFGC actually represents all cDNA clones used in all of the AFGC arrays including the 11 k, 13 k, and 16 k arrays.

### 2.2. Arabidopsis data preprocessing

We obtained the probe set ID, the vendor's corresponding mapping to AGI ID (for Arabidopsis arrays), and the nucleotide sequences of the probe sets (Table 1) directly from the vendors.

**Figure 1:**
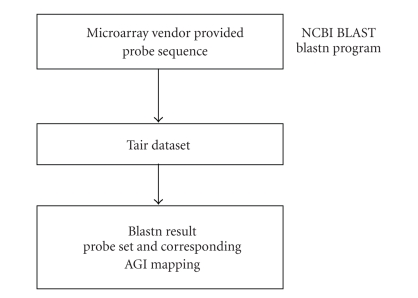
CCPMT *Arabidopsis* BLAST workflow. The workflow in CCPMT to get the probe set to AGI mappings is shown.

 In the case of *Arabidopsis*, all vendors provided the mappings between their probe sets and the corresponding AGI gene identifiers. However, due to evolving genome annotation, we derived a new set of mappings between the probe sets and the corresponding AGI IDs. The steps of the process are illustrated in Figure 1. The mapping was accomplished using the NCBI blastn [5] program. Blastn compares a nucleotide query sequence against a nucleotide sequence database. We used two different databases for blastn analysis. For the Affymetrix and Operon probe sequences, which do not contain introns, the AGI CDS database at TAIR was used as the sequence database due to the lack of introns and the UTRs in this database. The AGI CDS dataset is based on the TAIR6.0 release version, and was released in November 2005. For the AFGC and CATMA arrays, which do contain some intronic and UTR sequences, the AGI Transcripts dataset was used. The AGI Transcripts dataset includes all of the coding sequences from *Arabidopsis,* as well as containing the UTRs. Neither database contained intronic sequence. The AGI Transcripts dataset used the TAIR6.0 release version and was released in November 2005. The blastn expected value and percent identity cut-off were 10^−4^ and 98%, respectively.

### 2.3. Poplar data preprocessing

**Figure 2:**
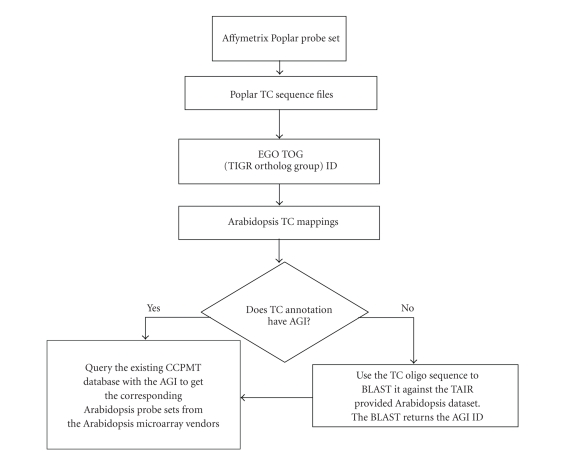
*Poplar*-*Arabidopsis* mapping. The above workflow explains the steps that were undertaken while mapping the Affymetrix *Poplar* probe set ID with the *Arabidopsis* probe set ID. TIGR EGO ID was used to go across species during mapping.

About 27% of the *Poplar* sequence have significant homology to *Arabidopsis* protein-coding sequences [6] and have been sequenced. Unlike *Arabidopsis*, *Poplar* does not have a universal gene annotation ID; so in CCPMT *Poplar,* probe sets are mapped within the species using the TIGR TC IDs and across plant species using the EGO database. The *Poplar* target sequences were sequence-aligned with the TIGR *Poplar* TC dataset using the blastn program as shown in Figure 2. The blastn expected value and percent identity cut-off were 10^−4^ and 98%, respectively. TIGR also provides a file with a mapping of the EGO ID and the corresponding TCs for all species. From this file, the mappings between EGO IDs and the corresponding *Arabidopsis* and *Poplar* TCs were parsed. The mapping of the TC to EGOs was assumed to be correct. In the future, any plant species with genes mapping to an EGO ID can be easily incorporated into CCPMT. Mapping the *Arabidopsis* TCs to their corresponding AGI IDs was achieved by using the *Arabidopsis* TC sequences (TIGR provides this file) and sequence-aligning with the TAIR “AGI Transcripts” dataset using blastn. Based on the cut-offs used there is the one-to-many mapping at several stages. A probe set can map to multiple genes, and multiple probe sets can map to one gene (Table 2).

**Figure 3:**
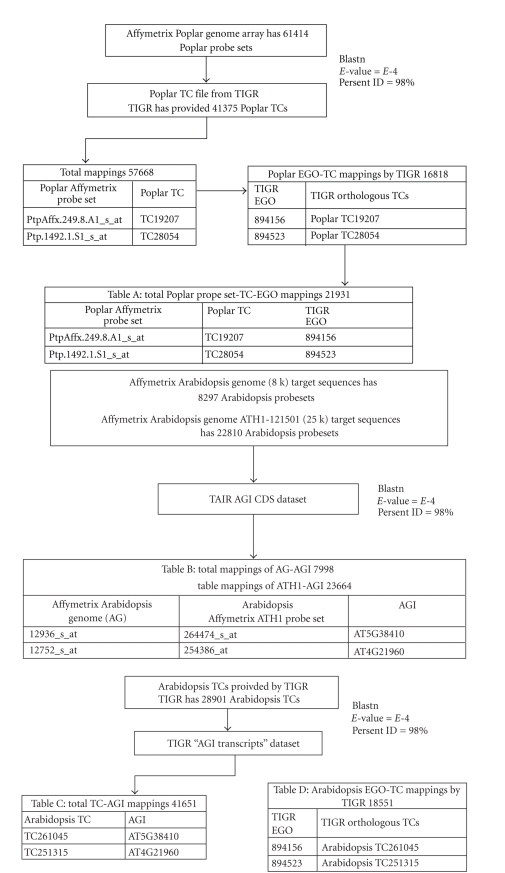

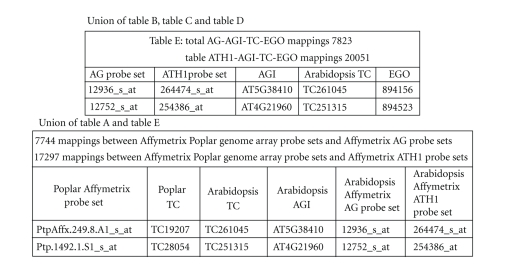
Workflow for the mapping between Affymetrix *Poplar*, Affymetrix AG, and Affymetrix ATH1 arrays.

As an example, Figure 3 illustrates the mapping of the Affymetrix *Poplar* Genome Array with the Affymetrix AG and Affymetrix ATH1 arrays; similar processes are used for the other arrays. Table 3 contains the number of matches that were found between all possible matches among arrays.

### 2.4. The CCPMT application

The CCPMT (http://www.ssg.uab.edu/ccpmt/) is composed of three pieces, namely, web pages (front end), core methods, and database (back end). The CCPMT web pages are written in JSP. Once the user hits the submit button, all of the data that have been entered are sent to the core code of Java servlets. The servlets act as the core methods that process the information received from the JSP pages and query the database. MySQL is used as the back-end database to store the microarray mappings. The code underlying the CCPMT is available from the corresponding author by request.

### 2.5. Using the CCPMT

The CCPMT is designed to be flexible and to allow for linking probes across arrays from a variety of starting data. CCPMT can be queried either at the probe set level or with identifiers such as the probe set IDs, AGI IDs, TIGR EGO IDs, or TC IDs, and output can be and is returned in these formats as well. As CCPMT is a web application, users can type or paste their queries in a textbox and, upon submission of the queries, the results are displayed in a browser-friendly format. One can also compare entire arrays by selecting the input array and the output array from the drop-down menu.

### 2.6. Example of the use of CCPMT

We illustrate the utility of the CCPMT via mapping the probe set 244904_at that is found on the Affymetrix AG array to determine which probe sets on the ATH1 array query the same gene. Step 1 (illustrated in Figure S1 in Supplementary Material available online at doi:10.1155/2008/451327) shows that the user wants to map the input data using Affymetrix probe set IDs. In addition, users' email address is entered so that the results can also be sent as an attachment in comma-separated file format. The next step (see Figure S2) is to enter the probe set(s), 244904_at in this case, and the species of the probe set, and to indicate which arrays to find homologous probe sets (in this example, Affymetrix AG and Affymetrix ATH1 arrays). The results are then displayed in Figure S3 which shows that the probe set 244904_at was mapped to 244922_s_at and 244923_s_at through the respective AGI IDs and that they map to AT2G07674.

## 3. DISCUSSION

Microarrays are gaining popularity in plant research. In addition, the requirement of many journals to deposit microarray data into public databases has made large amounts of data available for other investigators to use. But because there are a large number of arrays and array types, it can be difficult to compare data across datasets. We developed the CCPMT to allow investigators to identify common elements between databases rapidly and accurately.

While most vendors provide some mapping of probes to genes, in many cases the annotation is out of data or the companies use different standards for mapping. In some cases, there is considerable difference between our mapping and those provided with the arrays. This is due to at least three reasons. The first is that sequence, gene models, and annotation, especially for the incompletely sequenced genomes, can change rapidly. As a result, the provided annotation may be out of date. For example, data for CATMA and AFGC, obtained with TAIR at ftp://ftp.arabidopsis.org/home/tair/Microarrays/, had a timestamp of January 2006, but the FASTA file format has a timestamp of April 2004. The second reason for differences would be the choice of cut-off for mapping. We used >98% and E score of less than 10^−4^ for all but the AFGC arrays. Our choice of >98% is debatable, and somewhat different answers are obtained if other values are used; 98% may identify some paralogous genes, especially across species. It has not been conclusively established what level of sequence similarity is needed between a gene and a probe set for efficient binding. It is known that a single-base-pair difference in a short oligo can (with >50% of the time depending on the position of the SNP) destroy most binding. But since Affymetrix arrays usually have 11 sets of short oligos, the nonbinding of a single probe may or may not affect the overall RNA quantitation [7]. Long oligos bind relatively well with a few (1–3 bp) differences, but there is usually no redundancy of the addition of probes. cDNA clones can be quite long and only a portion of the sequence needs to be homologous for binding. A third source of difference may result from the choice of common genes. We used the TIGR EGO, but the NCBI HomoloGene (http://www.ncbi.nlm.nih.gov/sites/entrez?db=homologene) also identifies homologous genes across species. Unfortunately, these databases give slightly different mapping. We have used TIGR EGO database as it has more plant sequence data and has plant biologists devoted to curating the databases, as opposed to HomoloGene which is mammal-centric. Thus, the choice we made about cut-offs is conservative, but we have probably missed some probes with lower homology that actually do bind certain RNAs, and many others identify paralogous genes. As a result of these issues, our mapping is different from those provided by the vendor. The highest overlap is between the mapping provided by Affymetrix and the CCPMT mapping for the Affymetrix ATH1 array at 89%, while the AFGC has the lowest overlap at about 66%.

We think that the function allowing direct comparison of complete arrays is very useful for several reasons. One of the reasons why we developed the CCPMT was to allow coexpression analysis across arrays and species. This mapping in the CCPMT will be the basis of our next additions to CressExpress (http://www.cressexpress.org/), and others may use this as well for similar projects. Data from experiments that are often collected across time and different array platforms are used, which requires the mapping of probes across array platforms. This ability will be greatly amplified by the ability of the CCPMT to map data across platforms.

The annotation and sequence for genes as well as gene models are continuing to evolve, especially as additional species are sequenced. We have set up the CCPMT to allow for us to rapidly change the various portions of the database and mapping as data change. We plan to revise the CCPMT based upon new genomic information.

CCPMT currently has six *Arabidopsis* microarray arrays and one *Poplar* microarray. The tool was designed in such a way that one can easily incorporate a new microarray vendor for the current plant species as well as for new plant species. In the near future, we will rule out mapping for all Affymetrix-provided arrays for plant species, as well as those long oligo arrays from Operon and Agilent.

## Supplementary Material

Supplemental figures illustrate, via screen shots, the steps to use the CCPMT and an example of the output. Supplemental figure 1 shows a screen shot of the first step where the input data type, email address, and arrays to be searched are selected. Supplemental figure 2 shows a screen shot of the next steps where the data to be mapped is entered. Finally supplemental figure 3 shows the *.html output of the tool.

## Figures and Tables

**Table 1:** Web pages from where plant microarray data were downloaded.

	Probe sequence file location	Vendor-provided annotation file location
Affymetrix AG	http://www.affymetrix.com/support/technical/byproduct.affx?product=atgenome1	http://www.affymetrix.com/support/technical/ byproduct.affx?product=atgenome1
Affymetrix ATH1	http://www.affymetrix.com/support/technical/ byproduct.affx?product=arab	http://www.affymetrix.com/support/technical/ byproduct.affx?product=arab
Operon	http://omad.operon.com/download/index.php	http://omad.operon.com/download/index.php
CATMA	ftp://ftp.arabidopsis.org/home/tair/Microarrays/CATMA/	ftp://ftp.arabidopsis.org/home/tair/Microarrays/CATMA/
AFGC	ftp://ftp.arabidopsis.org/home/tair/Microarrays/AFGC/	ftp://ftp.arabidopsis.org/home/tair/ Microarrays/AFGC/
Agilent	NA (do not provide sequence files)	http://www.chem.agilent.com/Scripts/ PDS.asp?lPage=37068
Affymetrix *Poplar* Genome Array	http://www.affymetrix.com/support/technical/ byproduct.affx?product=poplar	http://www.affymetrix.com/support/technical/ byproduct.affx?product=poplar

**Table 2:** Comparing microarray vendor and CCPMT mappings.

Type of match	Affymetrix AG	Affymetrix ATH1	Operon	CATMA	AFGC
Number of probes per array type	8297	22810	29954	24576	19108
Nil entries from vendor (no mapping for these probes)	141	250	936	2969	2823
Absent-vendor; present-blast	0	0	0	0	1
Present-vendor; absent-blast	850	930	2335	2990	10952
Many-vendor; one-blast	124	584	0	30	117
One-vendor; many-blast	338	896	480	408	368
Exact match	6932	20193	26138	19551	6413^a^
Percentage of the vendor mapping numbers	84%	89%	87%	80%	34%

**Table 3:** Summary table of the number of probes that are linked between the various arrays currently in the CCPMT from the array in row to the arrays in columns. The above and below diagonal elements are slightly different for the methods we used such as Blasn, and percent identity is not always reflexive.

	Affymetrix AG	Affymetrix ATH1	AFGC	Agilent	CATMA	Operon	Affymetrix *Poplar* Genome Array
Affymetrix AG	—	7828	12170	7018	7193	8361	7744
Affymetrix ATH1	7827	—	30066	19188	20521	24636	17279
AFGC	12171	30066	—	29622	26070	30509	17793
Agilent	7018	19188	29622	—	18563	21371	16913
CATMA	7192	20521	26070	18561	—	23082	16378
Operon	8362	24636	30509	21371	23081	—	17505
Affymetrix *Poplar* Genome Array	7744	17279	17793	16912	16378	17504	—
