# Supplementary figures and images for: Cross-Chip Probe Matching Tool: A Web-Based Tool for Linking Microarray Probes within and across Plant Species
Source: Int J Plant Genomics. 2008 Oct 21;2008:451327. doi: 10.1155/2008/451327 (PMC2570748; doi:10.1155/2008/451327)

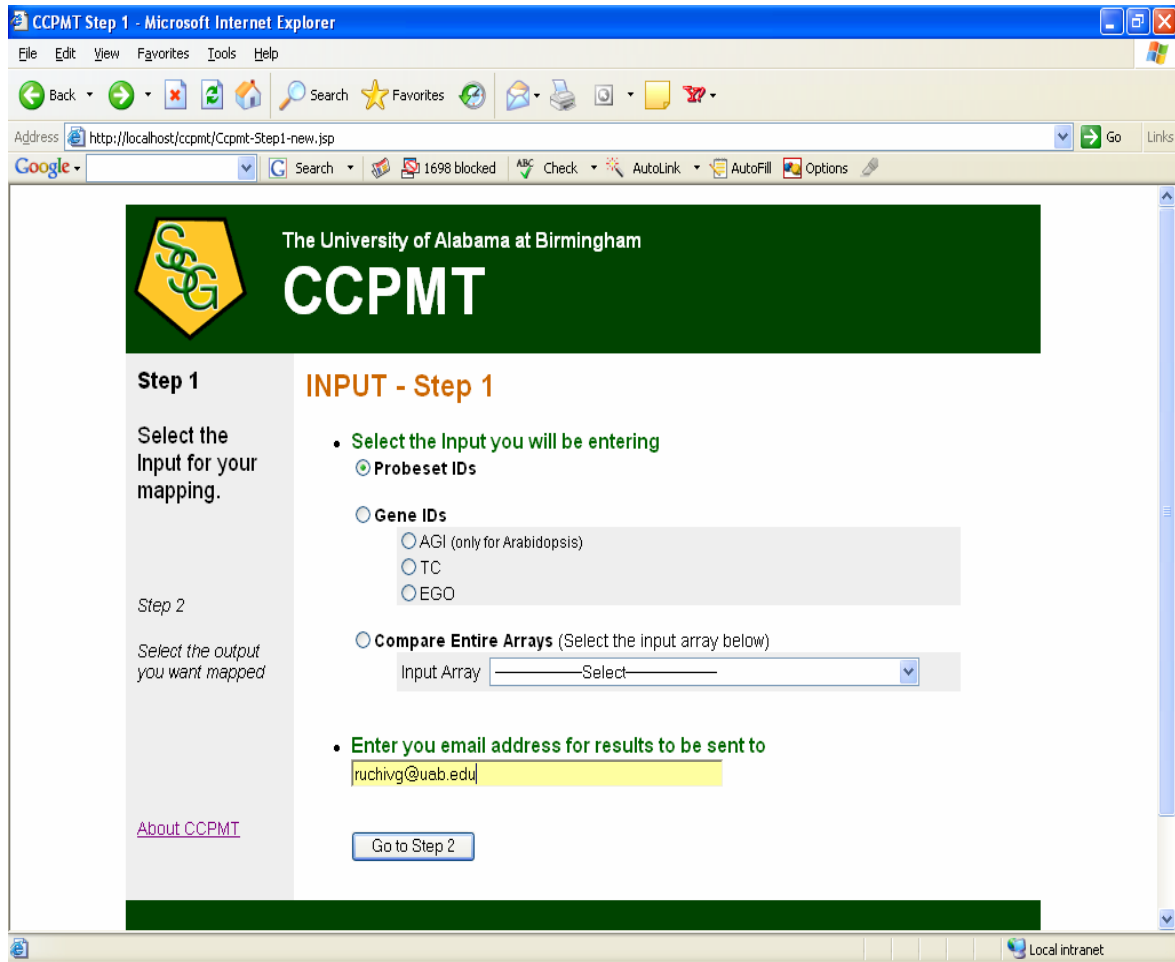

Supplement: Supplementary file 1 — Supplemental figures illustrate, via screen shots, the steps to use the CCPMT and an example of the output. Supplemental figure 1 shows a screen shot of the first step where the input data type, email address, and arrays to be searched are selected. Supplemental figure 2 shows a screen shot of the next steps where the data to be mapped is entered. Finally supplemental figure 3 shows the *.html output of the tool. [file 451327.f1.pdf]
