# Supplementary material for: Cross-Chip Probe Matching Tool: A Web-Based Tool for Linking Microarray Probes within and across Plant Species
Source: Int J Plant Genomics. 2008 Oct 21;2008:451327. doi: 10.1155/2008/451327 (PMC2570748; doi:10.1155/2008/451327)
Supplement: Supplementary file 2 [file 451327.f2.pdf]

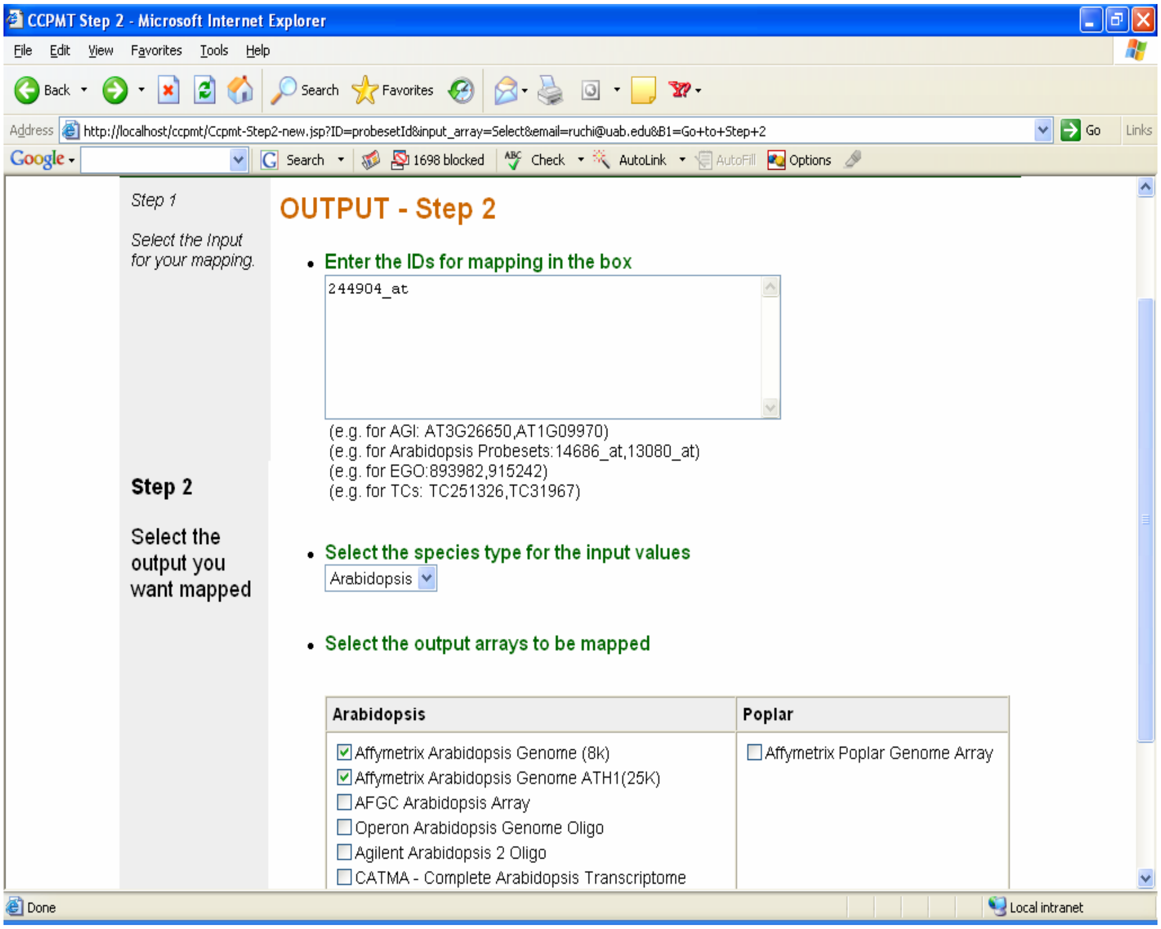
CCPMT Step 2 - Microsoft Internet Explorer
File Edit View Favorites Tools Help
Back Search Favorites
Address http://localhost/ccpmt/Ccpmt-Step2-new.jsp?ID=probesetId&input_array=Select&email=ruchi@uab.edu&B1=Go+to+Step+2
Go Links
Google Search 1698 blocked Check AutoLink AutoFill Options
Step 1
Select the Input for your mapping.
Step 2
Select the output you want mapped
OUTPUT - Step 2
Enter the IDs for mapping in the box
244904_at
(e.g. for AGI: AT3G26650,AT1G09970)
(e.g. for Arabidopsis Probesets:14686_at,13080_at)
(e.g. for EGO:893982,915242)
(e.g. for TCs: TC251326,TC31967)
Select the species type for the input values
Arabidopsis
Select the output arrays to be mapped
Arabidopsis
Poplar
Affymetrix Arabidopsis Genome (8k)
Affymetrix Arabidopsis Genome ATH1(25K)
AFGC Arabidopsis Array
Operon Arabidopsis Genome Oligo
Agilent Arabidopsis 2 Oligo
CATMA - Complete Arabidopsis Transcriptome
Affymetrix Poplar Genome Array
Done
Local intranet
