# Supplementary material for: Cross-Chip Probe Matching Tool: A Web-Based Tool for Linking Microarray Probes within and across Plant Species
Source: Int J Plant Genomics. 2008 Oct 21;2008:451327. doi: 10.1155/2008/451327 (PMC2570748; doi:10.1155/2008/451327)
Supplement: Supplementary file 3 [file 451327.f3.pdf]

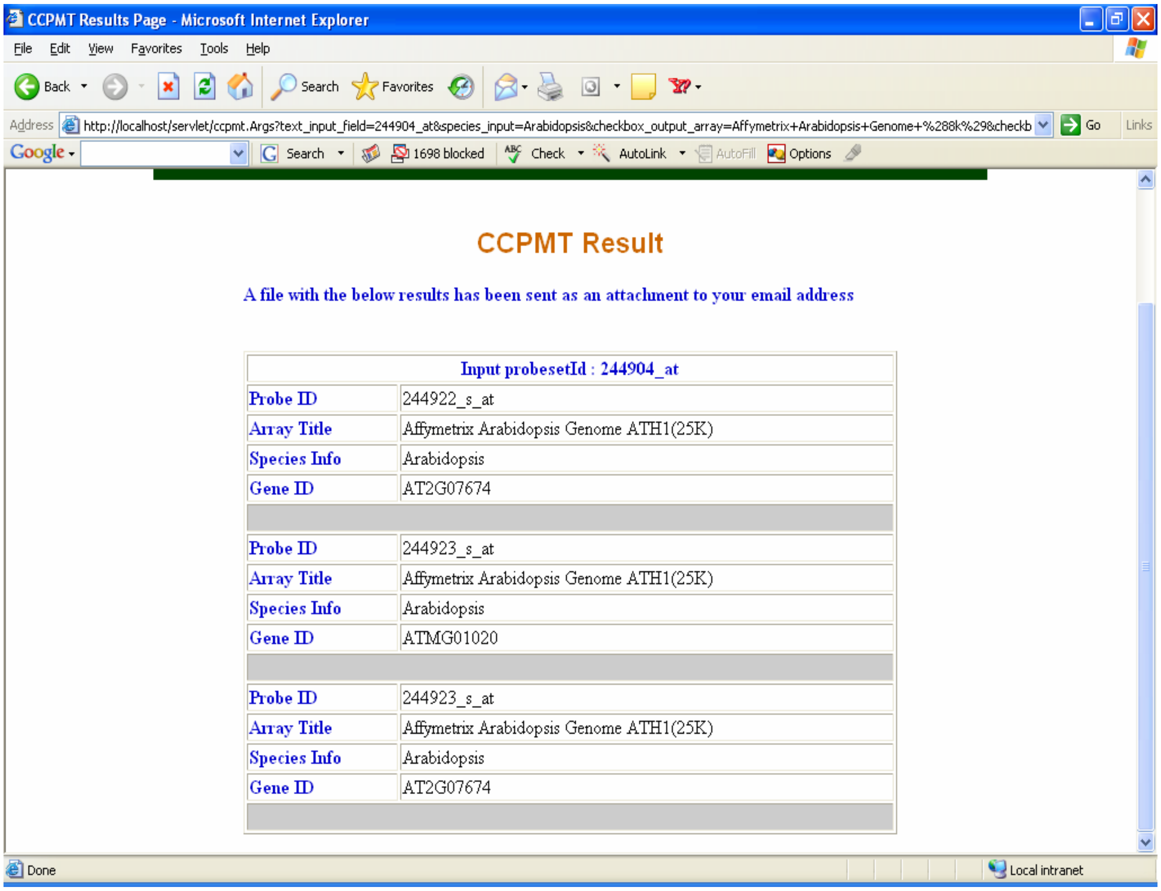

CCPMT Result

A file with the below results has been sent as an attachment to your email address

Input probesetId : 244904_at	
Probe ID	244922_s_at
Array Title	Affymetrix Arabidopsis Genome ATH1(25K)
Species Info	Arabidopsis
Gene ID	AT2G07674
Probe ID	244923_s_at
Array Title	Affymetrix Arabidopsis Genome ATH1(25K)
Species Info	Arabidopsis
Gene ID	ATMG01020
Probe ID	244923_s_at
Array Title	Affymetrix Arabidopsis Genome ATH1(25K)
Species Info	Arabidopsis
Gene ID	AT2G07674
